# PDHK-2 Deficiency Is Associated with Attenuation of Lipase-Mediated Fat Consumption for the Increased Survival of *Caenorhabditis elegans* Dauers

**DOI:** 10.1371/journal.pone.0041755

**Published:** 2012-07-27

**Authors:** Sunhee Kim, E-Jin Shin, Jeong-Hoon Hahm, Pil-Jong Park, Ji-Eun Hwang, Young-Ki Paik

**Affiliations:** Department of Biochemistry and Integrated Omics for Biomedical Science, World Class University Program, College of Life Science and Biotechnology, Yonsei Proteome Research Center, Yonsei University, Seoul, Korea; University of Geneva, Switzerland

## Abstract

In *Caenorhabditis elegans*, slow fat consumption has been suggested to contribute to the extension of the survival rate during nutritionally adverse conditions. Here, we investigated the potential role of pyruvate dehydrogenase kinase (PDHK)-2, the *C. elegans* homolog of mammalian PDK, effects on fat metabolism under nutritional conditions. PDHK-2 was expressed at low levels under well-fed conditions but was highly induced during long-term starvation and in the dauer state. This increase in *pdhk-2* expression was regulated by both DAF-16 and NHR-49. Dauer-specific induction of PDHK-2 was abolished upon entry into the post-dauer stage. Interestingly, in the long-term dauer state, stored fat levels were higher in *daf-2(e1370);pdhk-2* double mutants than in *daf-2(e1370)*, suggesting a positive relationship between PDHK-2 activity and fat consumption. PDHK-2 deficiency has been shown to lead to greater preservation of residual fats, which would be predicted to contribute to survival during the dauer state. A test of this prediction showed that the survival rates of *daf-2(e1370);pdhk-2(tm3075)* and *daf-2(e1370);pdhk-2(tm3086)* double mutants were higher than that of *daf-2(e1370)*, suggesting that loss of either the ATP-binding domain *(tm3075)* or branched chain keto-acid dehydrogenase kinase domain *(tm3086)* of PDHK-2 leads to reduced fat consumption and thus favors increased dauer survival. This attenuated fat consumption in the long-term dauer state of *C. elegans daf-2 (e1370);pdhk-2* mutants was associated with concomitant down-regulation of the lipases ATGL (adipose triglyceride lipase), HSL (hormone-sensitive lipase), and C07E3.9 (phospholipase). In contrast, PDHK-2 overexpression in wild-type starved worms induced lipase expression and promoted abnormal dauer formation. Thus, we propose that PDHK-2 serves as a molecular bridge, connecting fat metabolism and survival under nutritionally adverse conditions in *C. elegans*.

## Introduction

It has been well documented that a close relationship exists between energy metabolism and aging processes in animals, including the nematode *Caenorhabditis elegans* [1], [2]. In *C. elegans*, in particular, the critical role of insulin/insulin-like growth factor-1 (IGF-1) signaling (IIS) in metabolism and the control of aging has been well established. For example, suppression of the IIS pathway has been shown to cause a substantial increase in both mean lifespan (MLS) and fat accumulation [1]–[3]. This pathway also controls the dauer state, a non-aging condition that is important in determining the relationship between metabolism and aging [4], [5]. When *C. elegans* encounters unfavorable growth conditions (e.g., lack of food or high temperatures) or high concentrations of dauer pheromones (daumones or ascarosides), it can enter the dauer state [5]–[10]. Studies on dauer entry in *C. elegans* have suggested that this process is compartmentalized according to metabolic phase and growth time [11]. When L2 larvae prepare for dauer entry, nutritional adaptation accompanies intestinal and hypodermal fat accumulation, enabling *C. elegans* to survive an extended dormant period [12]. The dauer state is controlled by an IIS mechanism in which DAF-16 serves as a downstream effector [11]–[13].

The dauer state is resistant to environmental stresses, such as heat and cold, as well as oxidative stress, and the metabolic activity of dauer larvae is generally reduced [12]. Because dauer larvae do not feed, they use mostly stored triglycerides (TGs) as an energy source [12], [13]; TGs, in turn, are hydrolyzed to fatty acids by lipases [14]. Additional macronutrients that are stored and metabolized in dauer larvae include glycogen and trehalose [15]. Because their supply of stored nutrients is limited, dauers must suppress energy-consuming metabolic activities to survive [16]. Microarray and SAGE (serial analysis of gene expression) assays of dauer larvae, recovered dauers, and mixed-stage populations have suggested that the expression of enzymes involved in β-oxidation of fatty acids, glycolysis, glyoxylate, and gluconeogenesis pathways are enhanced under nutritionally adverse conditions, indicating that TGs are degraded by lipases and converted into sugars that are used for survival [17]–[21]. Slow energy release and tightly controlled energy consumption may be critical to long-term survival through diapause, hibernation, or long-term fasting in a variety of organisms via AMP-activated protein kinase (AMPK) function [14], [22]. In mammals, pyruvate dehydrogenase kinase-4 (PDK-4) is important in regulating pyruvate dehydrogenase during starvation [23]–[25].

In this study, we investigated how the pyruvate dehydrogenase kinase, PDHK-2, the *C. elegans* homolog of mammalian PDK-4 [23], contributes to the survival of *C. elegans* under the nutritionally adverse conditions. To this end, we used two different *C. elegans pdhk-2* mutants—*pdhk-2(tm3075)*, which lacks the C-terminal ATP-binding domain, and *pdhk-2(tm3086)*, which lacks the N-terminal mitochondrial branched chain α-ketoacid dehydrogenase (BCDH) kinase domain (Figure S1)—to assess changes in gene expression and enzymatic activity under the nutritional extremes of well-fed and 12-hour starvation, as well as in the dauer state. We then correlated these changes with regulation of fatty acid metabolism and dauer survival rate. Here, we report that PDHK-2 deficiency appears to contribute to the preservation of energy through down-regulation of the activity of a subset of lipases, namely ATGL (adipose triglyceride lipase), HSL (hormone-sensitive lipase), and C07E3.9 (phospholipase), under nutritionally adverse conditions, and thereby promotes the long-term survival of *C. elegans* in the dauer stage.

## Results

### PDHK-2 expression is highly induced in starvation and in the dauer state

**Figure 1:**
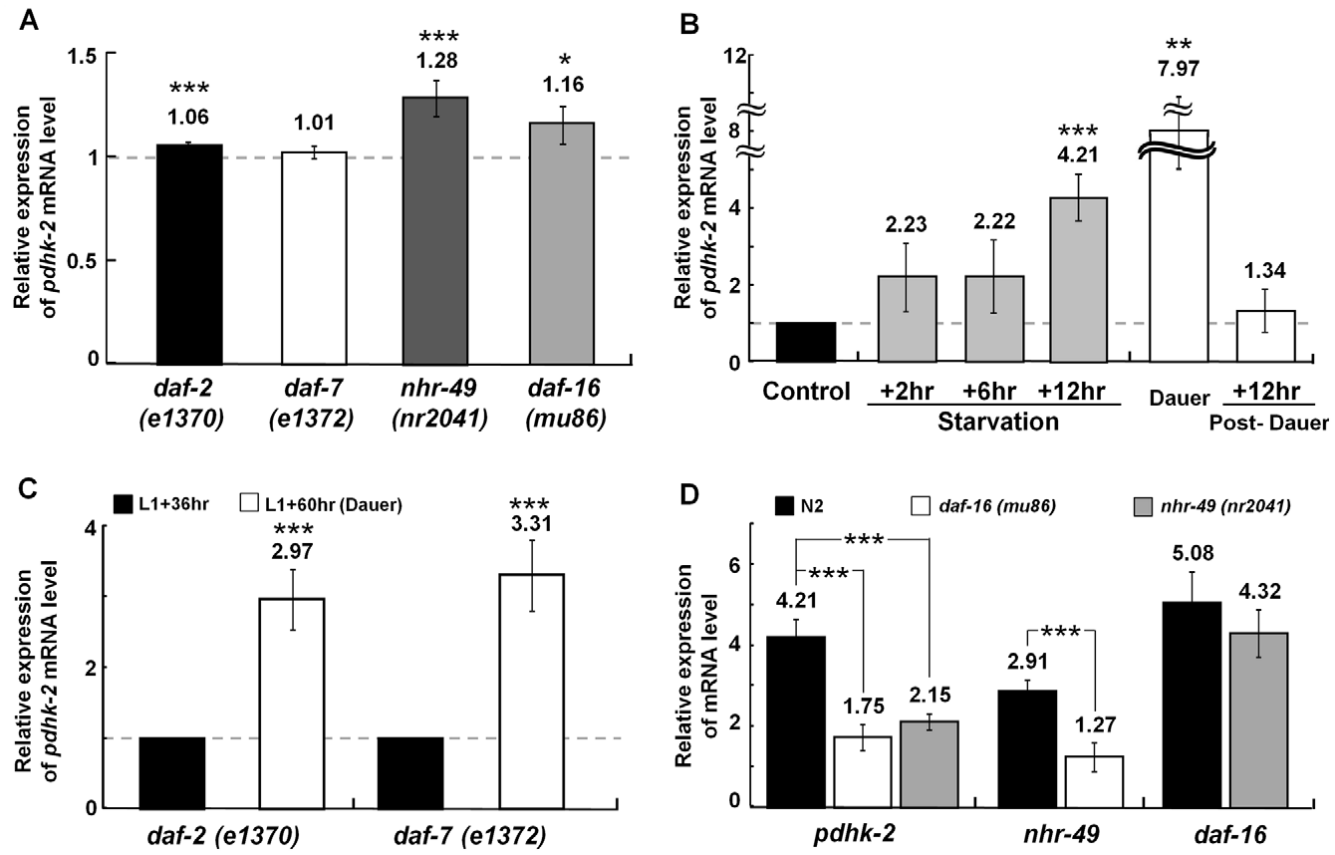
*pdhk-2* mRNA levels under various conditions. Changes in *pdhk-2* gene expression were determined by real-time qPCR. (**A**) Expression levels of *pdhk-2* in *daf-2(e1370)*, *daf-7(e1372)*, *nhr-49(nr2041)*, and *daf-16(mu86)* mutants from well-fed L4 stage worms. The data were normalized to N2 well-fed L4-stage worms. (**B**) Expression levels of *pdhk-2* in N2 worms starved for 2, 6 and 12 hours from the L4 stage, and in dauer and post-dauer stage worms. Dauer worms were obtained after starving for 7 days at 20°C, whereas post-dauer worms were produced by feeding dauer worms for 12-hour at 20°C. The values were normalized to well-fed conditions at the L4 stage worms. Worms maintained under well-fed conditions at the L3 stage were used as controls for the dauer stage group. (**C**) Expression levels of *pdhk-2* in *daf-2(e1370) and daf-7(e1372)* mutants in the pre-dauer (L1+36 hours) and dauer (L1+60 hours) states at 25°C. The values were normalized to pre-dauer (L1+36 hours) worms. (**D**) Expression levels of *pdhk-2*, *nhr-49* and *daf-16* in N2, *daf-16(mu86)* and *nhr-49(nr2041)* worms under 12-hour starvation conditions. In each experiment, mutant worms maintained under well-fed conditions at the L4 stage were used as controls for the starvation group. The values represent means [±SDs] from three independent experiments. *p<0.05, **p<0.01 and ***p<0.001 compared with the control.

To understand how PDHK-2 activity is related to longevity and fat metabolism, we first measured the MLS of two *pdhk-2* deletion mutants (Figure S2). In well-fed conditions, mutant strains showed no significant changes in MLS (*tm3075*, 20.5±1.4 days; *tm3086*, 19.5±1.0 days) compared to the wild-type N2 strain (20.5±1.8 days) (Figure S2A). Second, we measured fat density and TG content using Oil Red O staining and TG assays, respectively, and found no significant differences between N2 worms and the *pdhk-2* mutants, *tm3075* and *tm3086* (Figure S2B, C). Because DAF-2 and DAF-7 are known to play key roles in the IIS and tumor growth factor-β (TGF-β) signaling pathways, respectively, which are critical for the longevity and dauer formation, we also measured *pdhk-2* mRNA levels in *daf-2(e1370)* and *daf-7(e1372)* mutant worms using total mRNA prepared from L4-stage worms grown under well-fed conditions. We found that the expression level of *pdhk-2* did not significantly differ in *daf-2(e1370)* and *daf-7(e1372)* mutant worms compared to N2 worms (Figure 1A). Similarly, *pdhk-2* expression levels were unchanged in *nhr-49(nr2041)* mutants, which lack a nuclear hormone receptor involved in fatty acid metabolism, and *daf-16(mu86)* mutants, which lack the IIS downstream transcriptional regulator, DAF-16 (Figure 1A).

Because the expression of mammalian PDK-4 is known to increase during fasting [26], we examined changes in *pdhk-2* expression in N2 worms grown under starvation conditions. We found a substantial increase (4.2-fold) in *pdhk-2* gene expression in 12-hour starved groups compared to well-fed N2 worms (Figure 1B). Furthermore, *pdhk-2* expression was increased by 8-fold in the dauer state (induced by 7 days starvation of N2 on NGM plates at 20°C) compared to controls (Figure 1B). This dauer-specific induction of *pdhk-2* gene expression was abolished upon entry into the post-dauer stage (i.e., dauer recovery under well-fed conditions; Figure 1B). Similarly, as shown in Figure 1C, both *daf-2(e1370)* and *daf-7(e1372)* mutants also showed enhanced *pdhk-2* expression (2.9-fold and 3.3-fold, respectively) upon entry into the complete dauer state (L1+60 hours at 25°C; see ‘Materials and Methods’). This induction of *pdhk-2* expression in starvation and in the dauer state was not observed in either of the *pdhk-2* mutants, *tm3075* and *tm3086* (data not shown).

### PDHK-2 expression is regulated by DAF-16 and NHR-49 in the starved state

It has been previously reported that the mammalian F-box protein, FOXO1, the *C. elegans* homolog of DAF-16, induces PDK-4 expression in mammalian skeletal muscle during starvation [27]. Further, mammalian PDK-4 is known to be regulated by peroxisome proliferator-activated receptor (PPAR)-α, a functional homolog of *C. elegans* NHR-49 [28], [29] that is essential for the fasting-dependent induction of genes involved in converting fat stores into energy [30]. To investigate whether PDHK-2 is regulated by DAF-16 and/or NHR-49 in *C. elegans*, we measured *pdhk-2* mRNA levels in these mutants and N2 control worms that had been maintained under 12-hour starvation conditions. In contrast to the well-fed state, where mutant worms showed no significant changes in *pdhk-2* expression compared to N2 controls (Figure 1A), with long-term starvation, the relative expression levels of *pdhk-2* were more than 2.0-fold lower in *daf-16(mu86)* and *nhr-49(nr2041)* mutants than in N2 controls (1.7 and 2.1 for *daf-16(mu86)* and *nhr-49(nr2041)*, respectively, vs. 4.2 for N2; Figure 1D, left set of bars), indicating that both DAF-16 and NHR-49 act as activators of *pdhk-2* expression under long-term starvation conditions. To examine the epistatic relationship between DAF-16 and NHR-49 with respect to PDHK-2 activation, we measured the expression levels of *daf-16* and *nhr-49* mRNA in N2 controls and *daf-16(mu86)* and *nhr-49(nr2041)* mutant strains under 12-hour starvation conditions. Whereas the expression of *nhr-49* in *daf-16(mu86)* mutant was reduced compared to N2 with 12-hour starvation (1.2 vs. 2.9; Figure 1D, middle bars), the levels of *daf-16* expression in the *nhr-49(nr2041)* mutant were not significantly changed (Figure 1D, right bars, *p* = 0.1695). These results suggest that DAF-16 epistatically regulates NHR-49, while both DAF-16 and NHR-49 induce PDHK-2 under starvation conditions.

### Localization of PDHK-2 is changed during starvation

**Figure 2:**
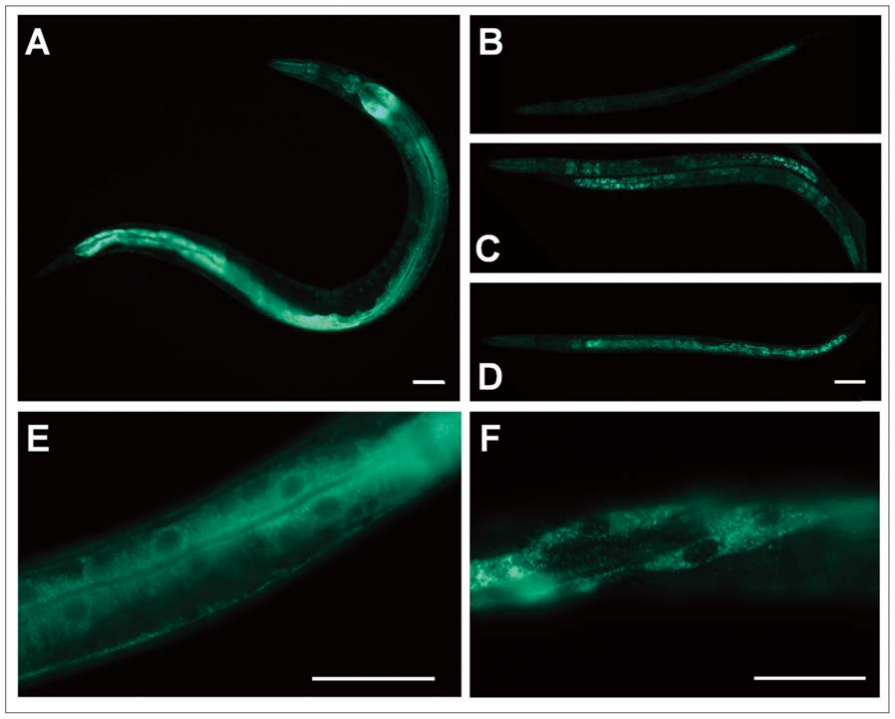
PDHK-2::GFP is highly expressed in starvation and dauer stages. (**A**) *pdhk-2*p::PDHK-2::GFP localization in the N2 adult stage. (**B**) *pdhk-2*p::PDHK-2::GFP localization in the L3 stage after good nutrition. (**C**) *pdhk-2*p::PDHK-2::GFP localization in the L3 stage after 12-hour of starvation. (**D**) *pdhk-2*p::PDHK-2::GFP localization in the dauer stage after 7 days of starvation. (**E**) Intestinal expression of *pdhk-2*p::PDHK-2::GFP under well-fed conditions at the L4 stage. (**F**) Muscle expression of *pdhk-2*p::PDHK-2::GFP in worms starved for 12-hour at the L4 stage. Scale bar: 50 µm.

To investigate if protein expression of PDHK-2 is changed by nutritional status, we first examined PDHK-2 localization in the well-fed state using the transgenic lines *pdhk-2* promoter::GFP (*pdhk-2*p::GFP) and *pdhk-2*p::PDHK-2::GFP (PDHK-2::GFP). We found that *pdhk-2*p::GFP (Figure S3) and PDHK-2::GFP (Figure 2A and Figure S4), respectively, are expressed mainly in the head neurons, pharynx, intestinal nuclei and intestine. The expression levels of both of *pdhk-2* mRNA and PDHK-2::GFP, which reflect PDHK-2 mRNA and protein expression, were increased under 12-hour starvation conditions (Figure 2; compare panel B with panel C) and in the dauer state (Figure 2; compare panel B with panel D). PDHK-2::GFP was ubiquitously expressed in the dauer state (Figure 2D). Interestingly, PDHK-2::GFP was dispersed around the nuclei of intestinal cells in L4 worms under well-fed conditions (Figure 2E). However PDHK-2::GFP exhibited a punctate fluorescent pattern in the muscle of worms that had been maintained under 12-hour starvation conditions (Figure 2F). These results suggest that localization of PDHK-2 is influenced by nutritional state (Figure 2E).

### PDHK-2 deficiency is associated with attenuated fat consumption and leads to increased survival rate of dauer larvae

Animals store fats in adipose tissue (intestines in *C. elegans*) primarily in the form of TGs, which are usually hydrolyzed by TG lipases [14]. Because it appears that the survival of *C. elegans* dauer larvae during the long-term dauer state heavily depends on the utilization of stored fat, the metabolic regulation of fats (lipolysis, mobilization, and utilization) is a key factor in determining survival rate [14]. Given the substantial induction (∼8-fold in starved N2) of *pdhk-2* expression in the dauer state, we investigated the potential role of PDHK-2 in the survival of worms in the dauer state using *daf-2(e1370);pdhk-2(tm3075)* and *daf-2(e1370);pdhk-2(tm3086)* double mutants. Our goal was to correlate changes in fat content, if any, with changes in the MLS of these mutants under well-fed conditions and in short-term (day 1) and long-term (day 10) dauer states.

**Figure 3:**
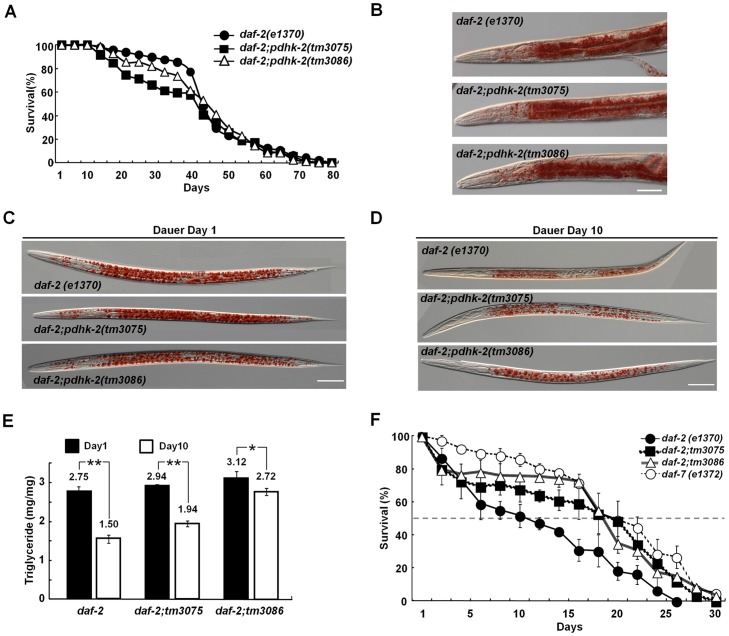
Functions of PDHK-2 in the dauer state. (**A**) Lifespan analysis of *daf-2(e1370)*, *daf-2(e1370)*;*pdhk-2(tm3075)*, and *daf-2(e1370);pdhk-2(tm3086)* mutant worms. (**B**) Oil Red O staining of *daf-2(e1370)*, *daf-2(e1370)*;*pdhk-2(tm3075)*, and *daf-2(e1370);pdhk-2(tm3086)* worms at the day 1 adult stage. (**C**) Oil Red O staining of *daf-2(e1370)*, *daf-2(e1370)*;*pdhk-2(tm3075)*, and *daf-2(e1370);pdhk-2(tm3086)* worms at the day 1 dauer stage. (**D**) Oil Red O staining of *daf-2(e1370)*, *daf-2(e1370)*;*pdhk-2(tm3075)*, and *daf-2(e1370);pdhk-2(tm3086)* worms at the day 10 dauer stage. (**E**) TG content in *daf-2(e1370)*, *daf-2(e1370)*;*pdhk-2(tm3075)*, and *daf-2(e1370);pdhk-2(tm3086)* worms at day 1 and day 10 of the dauer stage. The values represent means [±SDs] from three independent experiments. *p<0.05 and **p<0.001 compared with the control (**F**) Dauer survival assays performed on *daf-2(e1370)*, *daf-2(e1370)*;*pdhk-2(tm3075)*, *daf-2(e1370);pdhk-2(tm3086)*, and *daf-7(e1372)* mutant worms. Dauer worms were obtained from synchronized L1 stage worms after 60 hours at 25°C. The values represent means [±SDs] from five independent experiments. Scale bar: 50 µm.

First, in the well-fed state, as was previously observed in the *pdhk-2* single mutants (Figure S2A), there was no noticeable difference in MLS between *daf-2(e1370);pdhk-2(tm3075)* and *daf-2(e1370);pdhk-2(tm3086)* double mutants (40.1±2.1 and 44.5±1.8 days, respectively) and *daf-2(e1370)* control worms (45.5±1.3 days) (Figure 3A). Oil Red O staining assays also revealed no significant differences in fat density or TG quantity among the mutant strains tested (Figure 3B and Figure S2C). Moreover, neither dauer formation rates (at 20°C and 25°C) nor dauer recovery rates were different between *daf-2(e1370);pdhk-2* double mutants and *daf-2(e1370)* worms (data not shown).

Second, in the short-term dauer state, there was no significant difference in fat content between *daf-2(e1370);pdhk-2* double mutants and *daf-2(e1370)* control worms measured by Oil Red O staining (fats) and biochemical assays (TG levels) (Figure 3C, E), indicating that PDHK-2 may not be involved in fat metabolism in the early dauer state. However, in long-term dauers, there was generally more fat and fat granules were larger in *daf-2(e1370);pdhk-2*(*tm3075*) and *daf-2(e1370);pdhk-2(tm3086)* double mutants compared to *daf-2(e1370)* worms (Figure 3D, E), suggesting a close association between PDHK-2 and fat consumption in the late dauer state.

Third, measurements of fats remaining in the body of *daf-2(e1370)* controls and *daf-2(e1370);pdhk-2*(*tm3075*) and *daf-2(e1370);pdhk-2(tm3086)* double mutants revealed more residual TGs in the bodies of *daf-2(e1370);pdhk-2* double mutants (*tm3075*, 65.9%; *tm3086*, 87.1%) compared to *daf-2(e1370)* worms (54.5%). These results imply that fat consumption is significantly attenuated by a deficiency of PDHK-2 activity.

Lastly, we sought to determine how the amount of fat deposits remaining in the body of *daf-2(e1370);pdhk-2* double mutants influenced the survival rate of dauer larvae. We found that the survival rate, measured as 50% survival population (S_50_), was higher for *daf-2(e1370);pdhk-2(tm3075)* (15.4±1.88 days, *p*<0.01), *daf-2(e1370);pdhk-2*(*tm3086*) (16.5±0.97 days, *p*<0.001) double mutants and *daf-7(e1372)* (18.8±0.89 days *p*<0.0001) compared to *daf-2(e1370)* controls (11.4±1.76 days; Figure 3F). This increased survival rate, which ranged from 35% to 45%, appeared to be attributable to the higher levels of residual fat in the bodies of these double mutants, suggesting that both ATP-binding and BCDH domains may be associated with fat consumption. Thus, disruption of either leads to the reduced fat consumption that is necessary for survival during the long-term dauer state.

### PDHK-2 deficiency is associated with attenuated lipase activity

**Figure 4:**
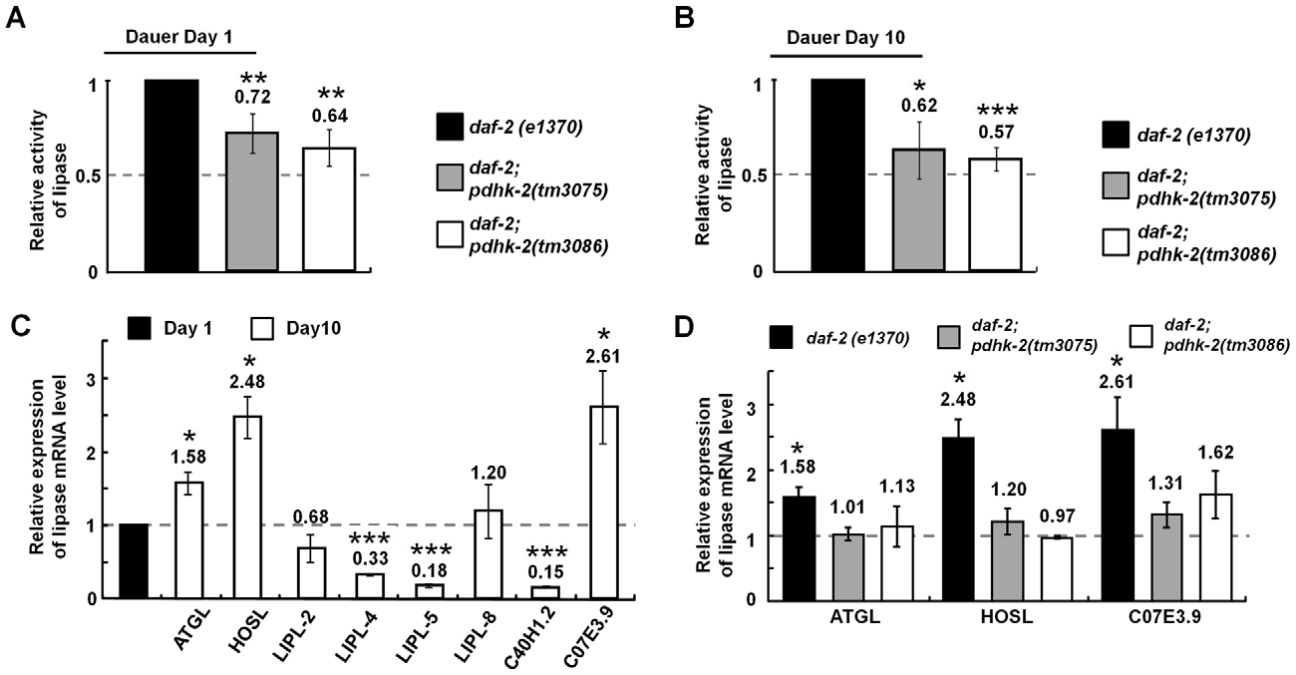
Expression pattern of lipases and their relative enzymatic activities in *daf-2(e1370)* controls and *daf-2(e1370)*;*pdhk-2 (tm3075) and daf-2(e1370);(tm3086)* double mutants. (**A**) Lipase activity of *daf-2(e1370);pdhk-2* double mutants at dauer day 1, normalized to *daf-2(e1370)* dauer worms. (**B**) Lipase activity of *daf-2(e1370);pdhk-2* double mutants at dauer day 10, normalized to *daf-2(e1370)* dauer worms. The values represent means [±SDs] from three independent experiments. (**C**) mRNA expression of lipases as determined by real-time qPCR in *daf-2 (e1370)* day 10 dauers. Data were normalized to *daf-2(e1370)* day 1 dauer worms. (**D**) Changes in lipase expression in *daf-2(e1370);pdhk-2* double mutants compared to *daf-2(e1370)* in day 10 dauers. Data were normalized to day 1 dauer worms. Dauer worms were obtained from synchronized L1 stage worms after 60 hours at 25°C. The values represent means [±SDs] from two independent experiments. *p<0.05, **p<0.01 and ***p<0.001 compared with the control.

Our results seem to suggest that PDHK-2 deficiency causes suppression of lipolysis, which is usually mediated by lipases. To test how lipase activity is influenced by PDHK-2 deficiency, we measured the enzymatic activity of total cellular lipases in the *daf-2(e1370)*;*pdhk-2*(*tm3075*) and *daf-2(e1370)*;*pdhk-2*(*tm3086*) double mutants at different dauer-state durations. In both short-term and long-term dauer groups, total lipase activity was substantially reduced in the double mutants (28–43%) compared to *daf-2(e1370)* controls (Figure 4A, B), confirming a strong positive correlation between lipase and PDHK-2 activity. There is very minor difference in lipase activity between the short-term dauer and long-term dauer of *daf-2(e1370)* (data not shown). Loss of either the ATP-binding (*tm3075*) or BCDH (*tm3086*) domain of PDHK-2 in *daf-2*;*pdhk-2* double mutants appeared to similarly contribute to this concomitant down-regulation of PDHK-2 and lipase activity (Figure 4A, B). To identify which lipase(s) might be involved in mediating fat catabolism in response to dauer state duration (short- vs. long-term), we examined the expression of eight currently identified and predicted lipases. We found highly induced expression of ATGL (1.6-fold), HSL (2.5-fold), and C07E3.9 (2.6-fold) in *daf-2(e1370)* long-term (day 10) dauers (Figure 4C). Notably, the increased expression of these three lipases was abolished in *daf-2(e1370);pdhk-2(tm3075)* and *daf-2(e1370);pdhk-2(tm3086)* double mutants during the long-term dauer state, indicating that these lipases may respond specifically to the suppression of PDHK-2 activity (Figure 4D).

### Overexpression of PDHK-2 induces abnormal dauer formation

**Figure 5:**
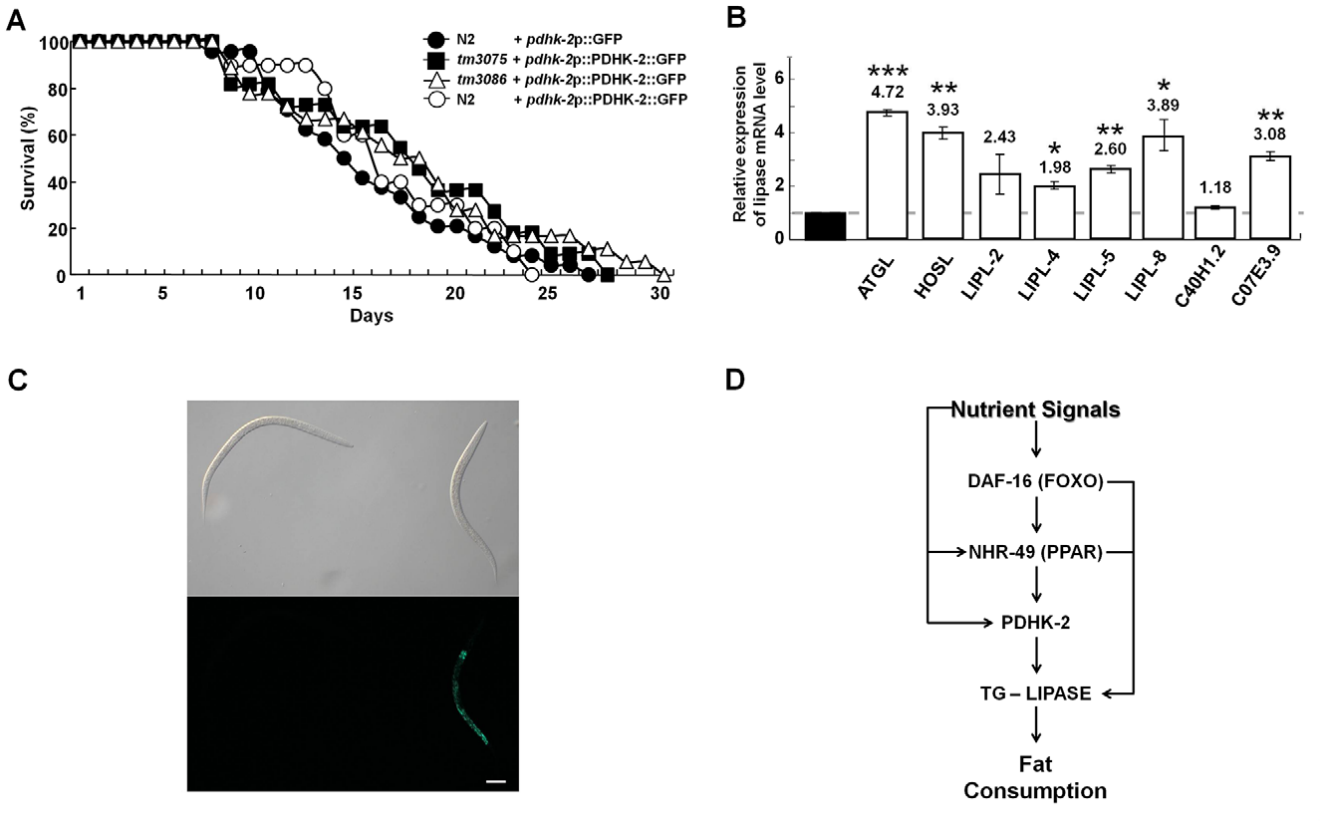
Effect of PDHK-2 overexpression. (**A**) Lifespan analysis of PDHK-2 overexpression in N2 wild-type. *pdhk-2*p::PDHK-2::GFP was microinjected into N2 worms and the *pdhk-2* mutants, *tm3075* and *tm3086*; N2 injected with *pdhk-2*p::GFP was used as a control. (**B**) Changes in lipase expression in PDHK-2-overexpressing N2 worms after 12-hour under starvation conditions. All data were normalized to control worms under 12-hour starvation conditions. *p<0.05, **p<0.01 and ***p<0.001 compared with the control. The values represent means [±SDs] from two independent experiments. (**C**) Effect of PDHK-2 overexpression in *daf-2(e1370)* dauer stage worms. In the lower panel, normal dauer worms (on the left) do not express GFP, whereas worms exhibiting abnormal dauer formation do express GFP (on the right). Scale bar: 50 µm. (**D**) Role of PDHK-2 under nutritionally adverse conditions in *C. elegans*. Shown is the proposed role of PDHK-2 in the maintenance of fat reservoirs in starved or dauer larvae, which depend on their fat supply for long-term survival. Nutrient signals act through DAF-16 and/or NHR-49 (or through a separate, currently unidentified mechanism) to regulate PDHK-2, which in turn appears to control the expression of lipases.

To investigate the effect of PDHK-2 overexpression (PDHK-2/OE) on the lifecycle of *C. elegans*, we microinjected *pdhk-2*p::PDHK-2::GFP into N2 worms and the *pdhk-2* mutants, *tm3075* and *tm3086*; N2 injected with *pdhk-2*p::GFP was used as a control. After growing under well-fed or starved conditions, we examined MLS and dauer formation rate. Well-fed worms showed no significant difference in MLS following PDHK-2 overexpression: N2 control (*pdhk-2*p::GFP), 16.4±2.1 days; N2;PDHK-2/OE, 17.7±2.0 days; *tm3075*;PDHK-2/OE, 18.5±1.5 days; and *tm3086*;PDHK-2/OE, 18.4±2.0 days (Figure 5A). In contrast, PDHK-2 overexpression in N2 caused an increase in the expression of most of lipases examined compared to control worms maintained under 12-hour starvation conditions (Figure 5B). Furthermore, when maintained under starvation conditions (>15 days at 25°C) or in daumone plate assays (data not shown) [31], *daf-2(e1370)* worms overexpressing PDHK-2 *(daf-2*(*e1370*;*PDHK-2/OE*) exhibited a reduction in the rate of dauer formation compared *to daf-2(e1370)* control worms (*daf-2(e1370);pdhk-2p::GFP*). That is, most *daf-2(e1370)* worms at the L1 stage typically entered dauer formation within 3 days (>60 hours) at 25°C, whereas transgenic *daf-2(e1370)*;PDHK-2/OE worms displayed slow growth, abnormal dauer formation, and even bypassed the dauer stage (Figure 5C). We also observed only a 15% survival rate when worms were subjected to 1% SDS treatment. Thus, PDHK-2 overexpression caused both an increase in lipase expression and abnormal dauer formation, although the relationship between these two events is not clear at present. It is also not clear whether overexpression of PDHK-2 caused the activation of lipases that consequently led to this aberrant dauer formation and lifecycle.

## Discussion

On the basis of the results presented here, we propose the following metabolic axis: PDHK-2 deficiency→decreased lipase-mediated fat consumption→increased survival rate under adverse nutritional conditions (i.e., dauer). This hypothesized relationship highlights an important regulatory role for PDHK-2 in starvation and the dauer state, where the glycolytic pathway has been shown to be less active [11], [13], resulting in the greater availability of free fatty acids as fuel. Considered in this context, our results suggest that, as long as the glycolytic pathway is active, fatty acid mobilization does not take place; accordingly, PDHK-2 would not appear to play any role under these circumstances. Our results further suggest that PDHK-2 is highly regulated by both developmental stage (i.e., dauer) and nutritional conditions, which affect PDHK-2 localization and protein distribution, as well as transcriptional activation. This latter effect is mediated by DAF-16 and NHR-49; thus, DAF-16 or NHR-49 deficiencies affect PDHK-2 activity under starved conditions. In contrast, PDHK-2 activity is unchanged in *daf-16(mu86)* or *nhr-49(nr2041)* mutants compared to *daf-2(e1370)* and N2 worms under well-fed conditions, consistent with the absence of a role for PDHK-2 in the context of an active glycolytic pathway.

Although PDHK-2 has previously been shown to contribute to longevity in *C. elegans* [32], [33], its role in MLS remains unresolved. For example, Kell et al. [32] reported that adult worms in which PDHK-2 was knocked down using RNA interference (RNAi) became sick or displayed vulval defects and exhibited shorter lifespans than control worms at 20°C. The authors concluded that the observed reduction in MLS caused by loss of PDHK-2 function might be the expected consequence, given that PDHK-2 plays an important role in energy consumption. However, Mouchiroud et al. [33] showed that inhibition or deficiency in *pdhk-2* expression increased MLS by 20% compared to control worms at 20°C. Our results clearly favor the latter of these two contradictory findings, but only in the survival rate of long-term dauers (Figure 3F), in which a PDHK-2 deficiency appears to attenuate fat consumption through concomitant suppression of at least three major lipases (ATGL, HSL, and C07E3.9), leading to increased the survival rate of dauer larvae. As our results confirmed that PDHK-2 can be regulated by nutritional state, it may worth measuring the changes in lifespan of *pdhk-2* mutants induced by dietary restriction or different food sources (e.g., HT115 or HB101 of *E. Coli*) in the hope that we can see better some effect of these two factors on the longevity of *pdhk-2* mutant in the future.

PDHK-2::GFP expression driven by the *pdhk-2* promoter was mainly detected in head neurons (proximal rear side of IL-2 neurons) and the intestine (Figure 2, S3 and S4). The expression pattern of PDHK-2::GFP in head neurons was maintained in both the dauer stage and during starvation. It has been reported that PDHK-2 can control the nuclear localization of SKN-1, a representative regulator of dietary restriction in head neurons [32], [34]. This observation suggests a potential link between the expression of PDHK-2 and SKN-1 in the head neuron. Although most lipases are expressed in the intestine [35], ATGL and HSL are known to be expressed in the nervous system, including in head neurons (Wormbase). Our data also show that PDHK-2 deficiency caused a decrease in ATGL, HSL and C07E3.9 (Figure 4D), consistent with this previous report. Thus, it is reasonable to speculate that the neuronal localization of PDHK-2 may be linked to metabolic control through the nervous system. In addition, PDHK-2 localization appears to be highly induced in muscle when worms are in starvation condition (Figure 2F). Muscle is one of the most energy consuming tissues which contain highest number of mitochondria. However, we could not find any clear evidence as to the correlation with PDHK-2 and mitochondrial localization (data not shown). It is also possible that post-translational modifications of PDHK-2 (e.g., phosphorylation, acetylation or glycosylation) could change the localization of this enzyme according to the nutritional state. For example, the well-fed condition causes activation of IIS, which might consequently phosphorylate PDHK-2 or its binding proteins, leading to different localization of this protein.

A characteristic morphological feature of dauer larvae is the presence of high-density fat granules in the intestine, which are very important for long-term survival during the dauer stage. Although studies have shown that L2 worms store fats before entering the dauer state in order to survive, how mobilization of these fats is regulated during this dormant period remains largely unknown. Since fat utilization is critical for long-term survival during the dauer state or starvation, our data suggest that PDHK-2 may be involved in the fine-tuning of fat consumption by modulating lipase expression specifically in response to the adverse nutritional conditions, in which IIS and TGF-β signaling are inactive (Figure 1C) but DAF-16 is active. That is, under well-fed conditions, PDHK-2 activity does not seem to influence lifespan and fat content, regardless of the activity of major signaling pathways or effecter molecules (NHR-49, DAF-16). However, with long-term starvation or in the dauer state, DAF-16 and NHR-49 apparently work to up-regulate PDHK-2, which consequently stimulates major lipases (e.g., ATGL, HSL and C07E3.9), leading to a supply of fat-driven energy for survival (Figure 5D). Conversely, if PDHK-2 is defective, such as in *pdhk-2(tm3075)* (this report), lipase expression is reduced (through an unknown mechanism), thereby inhibiting fat lipolysis and leading to sustained maintenance of a fat energy source necessary for survival. If PDHK-2 is also defective in dauer stage, lipase activity would also be reduced. In case of *daf-2*, there was essentially no change in lipase activity between dauer day 1 and day 10 (data not shown), indicating that different types of lipases might become activated during the dauer maintenance period (Figure 4C). In particular, PDHK-2 appears to control the expression of ATGL, HSL and C07E3.9, which may cause lipase activity lower than control (Figure 4D).

One probable clue to the relationship between PDHK-2 deficiency and decreased lipase activity may be found in the intersection of AMPK signaling and lipases. Accordingly, as previously reported [14], the extended dauer state of *C. elegans* dauer larvae is maintained through the control of the LKB1/AMPK pathway, which suppresses signals that promote lipase activity, resulting in the slow release of hypodermal fat. This observation is similar to our demonstration that mutation of *pdhk-2* leads to reduced lipase activity and greater accumulation of fats in the body, which may contribute to the increased the survival rate of dauer larvae (Figure 3C–F). Whereas LKB1/AMPK signaling directly controls a specific target lipase (i.e., ATGL) [14], PDHK-2 appears to regulate a broad range of candidate lipases in the dauer state (Figure 4D) and exerts differential regulation of lipases depending on the nutritional condition (data not shown). This led us to speculate that there might be nutrition state-specific lipase regulation, as is the case for LIPL-4, which is closely associated with the autophagy process through which longevity can be controlled in germline-less animals [36]. This observation suggests the possible existence of additional systems for regulation of lipases in response to nutrient signals. For example, under conditions of dietary restriction, PDHK-2 appears to be associated with AMPK and other factors [33].

PDHK-2 deficiency, which causes the increased survival rate through suppression of lipase activity in dauer larvae, appears to have the opposite effect in non-dauer animals. Loss of germline and reduced *daf-2* signaling were shown to synergistically induce the lipase *K04A8.5* (LIPL-4) and decrease fat storage [35]. RNAi-mediated *K04A8.5* knockdown partially suppressed the longevity of *daf-2(e1370)*. In mammals, PDK-4 appears to be important in controlling diabetes and obesity [37], and its expression at the mRNA and protein levels is regulated by seasonal changes in hibernating animals, which require tight control of fuel selection. In *C. elegans* dauer larvae, fine-tuning of energy consumption is critical to the success of long-term survival through diapause. Similarly, the dauer state of *C. elegans* is regarded as a hibernating period in which accumulated fats are consumed to support survival throughout this non-aging state. PDHK-2 levels were dramatically increased in this dauer state, consistent with an essential role for PDHK-2 in dauer fat metabolism and survival.

Lipases were highly expressed in PDHK-2-overexpressing N2 worms and *daf-2(e1370)* mutants leading to abnormal dauer formation through interruption of fat regulation, which is critical for dauer entry (Figure 5B, C). Although PDHK-2 overexpression showed no evident effect in the well-fed state, it negatively influenced dauer formation rate, perhaps due to over-consumption of lipids by highly activated lipase activity, leading to abnormal arrest (i.e., aberrant dauer formation). Thus, we propose that PDHK-2 serves as a molecular bridge, connecting fat metabolism and survival in coordination with DAF-16 and NHR-49 under nutritionally adverse conditions in *C. elegans* (Figure 5D).

## Materials and Methods

### Worm strains

The *C. elegans* strains used in this study, N2 Bristol (wild type), *daf-2*(*e1370*), *daf-7*(*e1372*) and *daf-16(mu86)*, were obtained from the *Caenorhabditis* Genetics Center. *nhr-49*(*nr2041*) was kindly provided by Carl Johnson and Nemapharm Pharmaceuticals. *pdhk-2*(*tm3075*) and *pdhk-2*(*tm3086*) were provided by the Japanese National Bioresource Project and were outcrossed six times to a wild-type background. Worms were cultured on nematode growth media (NGM) agar plates seeded with the *Escherichia coli* strain OP50, as previously described [38]. *daf-2*(*e1370*);*pdhk-2*(*tm3075*) and *daf-2*(*e1370*);*pdhk-2*(*tm3086*) double mutants were obtained through genetic crosses.

### Transgenic lines and plasmid construction

The *pdhk-2* promoter::green fluorescent protein (GFP) construct (*pdhk-2*p::GFP) was produced by cloning a 2.5-kb sequence upstream of the *pdhk-2* start codon into the pPD95.75 vector using standard molecular biology protocols. The *pdhk-2*p::PDHK-2::GFP construct contained a 1.2-kb *pdhk-2* cDNA sequence (immediately prior to the stop codon) between the promoter sequence and GFP in the pPD95.75 vector. Constructs were microinjected at 50–100 ng/µl into N2, *daf-2(e1370)*, and *pdhk-2* mutant worms. GFP-expressing transgenic worms were selected from the progeny and analyzed.

### Starvation assay

Starvation assays were performed as described previously [30]. For starved worms, embryos from bleached adults were allowed to hatch in S-basal medium. L1 larvae were added to NGM plates containing OP50 (well-fed condition). Worms were washed at the L4 stage using distilled water and placed onto assay plates (NGM without peptone) containing no bacteria for 2, 6 or 12 hours.

### Quantitative RT-PCR

Total RNA was isolated from worms at various stages using the illustra RNAspin Mini RNA Isolation Kit (GE Healthcare). Total RNA was reverse transcribed with the Transcriptor First Strand cDNA Synthesis Kit (Roche) using oligo (dT) primers. Quantititative polymerase chain reaction (qPCR) was performed using an MJ Research Chromo4 Detector with the QuantiTect SYBR Green PCR kit, as described by the manufacturer (Qiagen). β-actin was used as an internal control. The data are presented as means (± standard deviations) of triplicate reactions from two or three independent experiments.

### Lifespan assay

Lifespan assays were performed at 20°C under well-fed conditions. Embryos from bleached adults were allowed to hatch in S-basal medium to obtain highly synchronous L1 stage animals. L1 worms were then transferred to NGM plates and allowed to mature to the adult stage. Adult worms were transferred to fresh NGM plates each day during the period of reproduction. Worms that no longer responded to gentle prodding with a platinum wire were considered non-viable.

### Oil Red O staining

Worms were stained with Oil Red O as described previously [39]. Worms were grown on NGM plates until the day 1 adult stage and then were washed three times with 1× phosphate-buffered saline (PBS). Worms were resuspended in 1× PBS and 2× MRWB (Modified Ruvkuns Witches Brew) buffer with paraformaldehyde for 1 hour at room temperature. In the case of dauer worms, additional freeze/thaw steps were performed using liquid nitrogen. Worms were washed with 1× PBS and then resuspended in 60% isopropanol and incubated for 15 minutes at room temperature. Isopropanol was removed, 60% Oil Red O stain was added, and worms were incubated overnight with rocking. Worms were washed with 1× PBS containing 0.01% Triton X-100 and visualization by microscopy.

### TG determination and lipase assay

Proteins from well-fed and dauer worms were harvested as described previously [40]. Day 1 adult or dauer worms were washed three times with ice-cold M9 buffer. Worms were then flash frozen in liquid nitrogen without buffer. Approximately 100–200 µl of worms were ground in a nitrogen-chilled mortar and pestle, then gathered in a reaction tube and kept on ice. PBS (1×) was added to the powdered worms, and the extracts were sonicated and then centrifuged for 7 minutes at 12,000×*g* and 4°C to remove any insoluble matter. Protein concentration was measured by the Bradford assay. TG content was determined using a commercially available TG determination kit (Sigma-Aldrich). Lipase activity was determined using a QuantiChrom Kit (BioAssay Systems).

### Dauer survival assay

Dauer worms were obtained by growing synchronized L1 stage worms (*daf-2(e1370)*, *daf-7(e1372)* or *daf-2;pdhk-2* double mutants) on assay plates (seeded with 2 ml OP50 culture) for 60 hours at 25°C. Dauer larvae thus obtained were washed five times with distilled water and diluted to 500 dauers/10 µl. A 500 µl suspension of dauer larvae was mixed in a 360° rotator (30 rpm) at 20°C so that dauer may continuously consume energy for survival. The five micro liter (5 µl) aliquots of dauer larvae suspension were spotted on the petri dish (>10 spots). Those worms showing L4 stage (exit from the dauer stage) and dead worms were removed from the plate and excluded from worm counting (usually 1–2%). About 5 µl aliquots of dauer larvae suspension were treated with 1% SDS and survival rate was determined 1 h after SDS treatment. Values represent the means (± SDs) of each test, which was repeated five times. For the statistical analysis, we used student's t-test which has been used previously [41]. For most of data point, p values are present in a conventional way (from the larger to the smaller values).

## Supporting Information

Figure S1 **Structure of the** ***pdhk-2*** **gene.** The *pdhk-2* gene (indicated by the black box) is located on chromosome III in *C. elegans*, and is comprised of 8 exons and 7 introns. The gray boxes denote exons while white boxes, black lines, and dotted lines represent translated regions, introns, and deleted segments, respectively. Mutant *tm3075* has a deletion that encompasses regions of exon 6, intron 5, and part of intron 6, which correspond to a portion of the ATPase-like ATP binding region and the histidine kinase motif. Mutant *tm3086* has a deletion in exons 3 and 4, as well as and introns 2 and 3, which are translated into mitochondrial branched-chain alpha ketoacid dehydrogenase kinase.
(TIF)

Figure S2 **Lifespan and fat content of** ***pdhk-2*** **mutants under well-fed conditions.** (**A**) Lifespan analysis of N2, *pdhk-2(tm3075)*, and *pdhk-2(tm3086)* worms. (**B**) Oil Red O staining in day 1 adults. (**C**) Triglyceride content in N2, *pdhk-2(tm3075)*, *pdhk-2(tm3086), daf-2(e1370)*, *daf-2*;*pdhk-2(tm3075)*, and *daf-2;pdhk-2(tm3086)* worms at day 1 adults. Three independent experiments were performed. Error bars indicate the standard deviation.
(TIF)

Figure S3 **Expression pattern of the PDHK-2::GFP in** ***C. elegans*** **.** A PDHK-2/GFP fusion construct was microinjected into N2 worms. (**A**) and (**B**), GFP-tagged PDHK-2 promoter is shown to be expressed in the head, intestine and the nuclei of intestinal cells. (**C**) and (**D**), *pdhk-2*p::PDHK-2::GFP expressed in the head and intestine. Both constructs were microinjected into worms at 50–100 ng/µl. Scale bar: 50 µm
(TIF)

Figure S4 **Expression of PDHK-2 in the head neurons of JK2868 dauer stage worms.** *pdhk-2* p::PDHK-2::red fluorescent protein constructs were microinjected into JK2868 worms at 50–100 ng/µl. Dauer worms following 7 days of starvation at 25°C. Red indicates *pdhk-2* p::PDHK-2::red fluorescent protein and green corresponds to *lag-2* p::GFP. Scale bar: 50 µm.
(TIF)
